# Supplementary figures and images for: Leveraging foundation models to dissect the genetic basis of cluster compactness and yield in grapevine
Source: Sci Rep. 2025 Dec 6;16:1434. doi: 10.1038/s41598-025-31531-y (PMC12795812; doi:10.1038/s41598-025-31531-y)

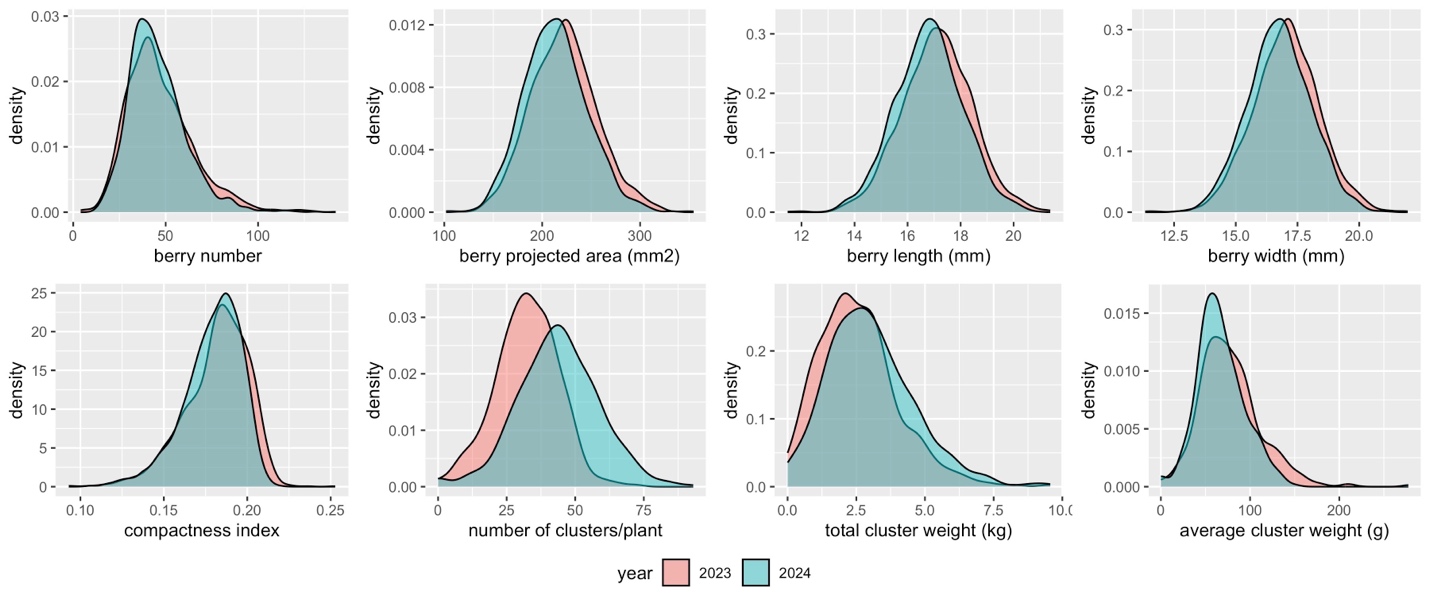


Supplementary Figure 1. Raw data distribution of all the traits measured across both years (2023 and 2024).

Supplement: Supplementary file 4 — Supplementary Material 4 [file 41598_2025_31531_MOESM4_ESM.docx]
